# Referral and utilization patterns for home-based palliative care services among older adults

**DOI:** 10.1186/s12904-026-02179-w

**Published:** 2026-06-11

**Authors:** Joohyun Chung, Aaron Langlois, TylaAnn Burger, David L Chin

**Affiliations:** 1 Elaine Marieb College of Nursing, University of Massachusetts Amherst, 224 Skinner Hall651 North Pleasant Street, Amherst, MA 01003 United States; 2 Palliative & Hospice Care, VNA Care, Worcester, MA United States; 3 School of Public Health & Health Science, University of Massachusetts Amherst, Amherst, MA United States

**Keywords:** Home-based palliative care, referral patterns, equity in palliative care

## Abstract

**Background:**

Home-based palliative care (HPC) improves quality of life for individuals with serious illness but remains underutilized in the United States, with persistent disparities in access. HPC is also frequently misconceived as appropriate only at the end of life.

**Aim:**

This study examined disparities in access to HPC by comparing patients who received services with those who were referred but not enrolled.

**Methods:**

A secondary analysis was conducted using 567 clinical records from a healthcare facility in the eastern United States between 2022 and 2023. Descriptive statistics, chi-square tests, and Cox proportional hazards regression model were used to compare demographic and clinical characteristics between groups.

**Results:**

No significant differences were observed by gender or race between patients who received HPC and those who did not. Significant differences were found for age (t = 2.33, *p* = 0.02), insurance type (*p* < 0.001), and referral source (*p* = 0.02). Patients who received HPC were more likely to be older, female, insured through Medicare Part B, and referred from larger hospitals.

**Conclusion:**

These findings underscore ongoing structural disparities in access to home-based palliative care and highlight the need for targeted strategies to improve equitable access to HPC services.

## Introduction

The aging population in the United States is rapidly increasing. Currently, one in six Americans is aged 65 or older, and this proportion is projected to rise to nearly one in four by 2060 [1]. Aging is associated with a higher risk of chronic illnesses such as dementia, heart disease, type 2 diabetes, arthritis, and cancer, as well as greater vulnerability to infections like influenza and pneumonia. Many older adults live with multiple chronic illnesses and complex care needs, creating a growing demand for care models that focus on quality of life, symptom relief, and support for patients and families [2]. Home-based palliative care (HPC) has emerged as a promising solution, offering interdisciplinary, patient-centered care in the home setting.

Despite the growing need for supportive care at home, a significant gap exists between older adults’ preferences and actual experiences. Although nearly 70% of older adults prefer to die at home, only about 30% do [3]. Importantly, these patterns reflect not only healthcare system constraints but also the fact that death is a deeply social experience shaped by family capacity, caregiving relationships, and the broader home environment. These patterns are often driven by the limited availability of home-based palliative and hospice services, inadequate funding, financial hardship for patients and families, as well as the significant caregiver burden placed on unpaid family members and other informal caregivers [4–7]. Home-based palliative and hospice care encompasses two distinct but related concepts: palliative care and hospice care. Both provide holistic support for serious illness. Palliative care can start anytime, even with treatment; hospice begins when treatment stops [8].

Palliative care delivers medical care designed for people living with a serious illness, addressing physical, functional, psychological, social, and spiritual needs while emphasizing personalized care for both patients and their families [9]. Home-based palliative care (HPC) refers to specialized palliative care services delivered in the patient’s home rather than in hospital or clinic settings. HPC is a form of community-based care that brings services directly to the patient’s place of residence, including private homes, assisted living facilities, and nursing homes [10, 11]. By providing care in the home environment, HPC reduces transportation and mobility barriers and improves access for individuals with advanced illness or functional limitations [12–14]. HPC programs vary in structure, staffing, and intensity. Depending on patient needs and available resources, care may be delivered by individual clinicians or interdisciplinary teams, with visit frequently tailored to clinical complexity. HPC has been identified as a preferred care model for many older adults due to its ability to support comfort, familiarity, independence, autonomy, and family involvement [10, 15].

Access to HPC is facilitated through formal referral pathways. Referrals are typically initiated by primary care providers or hospital-based clinicians and require eligibility assessment and coordination through outpatient or community-based palliative care services. In some healthcare systems, direct hospital-to-home referral pathways also exist. HPC is a specialized service that is distinct from primary care but is accessed through both primary and hospital-based healthcare systems. The effectiveness of HPC delivery depends on the completeness of referral processes, including transfer of clinical history and individualized care needs [16, 17]. However, coordination between hospital and home-based services remains fragmented, particularly in rural settings [16–18].

Evidence indicates that HPC is associated with reduced symptom burden, lower healthcare utilization and costs, improved care satisfaction, and a greater likelihood of dying in the preferred place of residence [4–7, 10, 19]. It is also associated with a higher likelihood of dying in one’s preferred place, most commonly the home. Despite these benefits, there remains limited understanding of HPC utilization among older adults, particularly regarding differences between those who are referred and engage in services and those who are referred but do not engage. To address this gap, this study examined differences between older adult patients who were referred to hospice and palliative care (HPC) services and subsequently received care, and those who were referred to but did not engage in services. The guiding research question was: What are the differences between older adult patients who are referred to HPC services and engage in care compared with those who are referred but do not engage?

## Methods

A quantitative, cross-sectional study design was utilized through secondary data analysis. The study was approved by the University of Massachusetts Amherst, Institutional Review Board (IRB No. 5098) and was determined not to involve human subjects. A fully de-identified dataset authorized for secondary analysis was used; therefore, patient consent was not required under HIPAA standards.

### Conceptual framework

Levesque’s healthcare access framework was used as the conceptual guide for interpreting study findings rather than as a fully operationalized analytic model. The framework identifies multiple barriers to healthcare access, which highlights challenges such as approachability, acceptability, availability, accommodation, affordability, and appropriateness [20]. Given the scope of the available data, this study does not directly measure all domains of the framework but focuses on selected factors related to access and utilization of home palliative care (HPC). This study primarily investigates factors such as gender, age, insurance, the source of the referral, whether they have completed a MOLST (Medical Orders for Life-Sustaining Treatment, refer to Methods: variables) form or Advance Care Plan, and the time between receiving a referral and accessing the first HPC service. It aims to demonstrate how these factors impact access to HPC. These elements significantly affect individuals’ health and behaviors and can particularly obstruct access to HPC among vulnerable older adults. These variables were selected as measurable indicators that may relate to specific dimensions of access, such as availability, accommodation, and affordability. Accordingly, the framework is used to contextualize and interpret observed patterns in access rather than to test all components of the model. This approach allows for a structured interpretation of how system- and patient-level factors may influence access to HPC among older adults while acknowledging that not all aspects of the framework are captured in the dataset.

### HPC (Home-based Palliative Care) setting

#### A brief overview of the facility

The palliative care program, which operates within a nonprofit home healthcare organization that has served the Eastern United States for over 130 years, has been providing care since 2010. It supports individuals with serious or complex health conditions at any stage of illness, with a focus on improving comfort and quality of life. The program primarily serves suburban and rural areas, including small towns and cities, and operates independently of hospice and curative treatments. 

#### Services provided

The palliative care program offers comprehensive, patient-centered services delivered in the comfort of patients’ homes. Key services include symptom management (addressing pain, fatigue, and other distressing symptoms); emotional and psychological support (counseling for patients and families); spiritual care (provided by chaplains); caregiver support (including education and respite care); and end-of-life planning (including advance care directives and comfort-focused care). Care is delivered through a combination of in-person visits and telemedicine and is tailored to each patient’s needs and preferences. Services are coordinated with an interdisciplinary team that includes primary care providers, palliative care physicians, nurses or nurse practitioners, social workers, chaplains, hospice aids, and trained volunteers to address medical, physical, emotional, practical, and spiritual needs. Services are provided to both patients and their families across the trajectory of serious illness. Care is available regardless of the patient’s place of residence, including private homes, apartments, nursing homes, or skilled nursing facilities. In some cases, hospice services may also be delivered in short-term inpatient settings, such as hospice residences.

#### Referral volume and trends

In 2023, the program received a total of 2,984 referrals, with 23% directed to palliative care and 77% to hospice care (which is managed by a different department/team). For our study, we focused exclusively on palliative care. From January 1 to December 19, 2024, the program received 2,835 referrals, with palliative care increasing to 27% and hospice care decreasing to 73%.

#### Patient transitions and outcomes

In 2023, 12% of palliative care patients died without transitioning to hospice care. This percentage improved in 2024, with only 9% of palliative care patients passing without hospice enrollment.

#### Hospital readmissions and workflow

Hospital Readmissions are not currently monitored. HPC consultations are focused on symptom management and do not involve monitoring of daily activities. Additionally, the facility does not track the workflow or the average number of visits per patient, which could be useful information to collect moving forward.

#### Funding structure

The palliative care program is funded through Medicare Part B, Medicare Advantage, and commercial insurers, using Physician and Master of Social Work (MSW) codes. Medicaid coverage is generally not available, except for patients referred to by a specific medical group. Additionally, not all commercial payers offer palliative care benefits. Payment is capped at two months of care per patient under a single physician group contract.

### Sampling and study participants

A total of 567 clinical data records were retrieved from a non-profit facility in the Eastern United States. The inclusion criteria were: (1) aged 65 or older, and (2) having received a referral for HPC to the facility from a hospital, primary care provider, or another healthcare source, regardless of whether they received HPC services from the facility. The exclusion criterion was individuals under the age of 65.

### Data and variables

De-identified data from the Electronic Health Records (EHRs) at a non-profit facility between October 2022 and September 2023 were retrieved for this study.

#### Demographic variables

Gender, age, insurance, referral types, referral date, and visiting date were included, except race, because race had more than 80% missing data. Referrals to the HPC can be initiated by various healthcare providers or care coordinators. In this study, the referral types variable is categorized into three groups, all originating from outside the facility: (1) hospitals or medical centers, where referrals are typically made by inpatient teams or hospital-based specialists; (2) primary care providers or physicians’ offices, where referrals are initiated by outpatient clinicians or family doctors; and (3) other sources, which include community-based care coordinators, other home health agencies, or other sources.

#### Reasons for not receiving HPC despite a referral

An open-ended question was posed to explore the reasons why a patient did not receive HPC care, despite having been referred. For this unstructured data, although the study primarily employs quantitative methods, we manually categorized the patients’ responses to identify common themes and reasons for not engaging in HPC services. For example, five categories were created to classify the responses: no clinical availability, no payor sources, refused, transferred/referred to others (with four subcategories: hospitalized before the first visit, referred to another home health agency, hospice, or Skilled Nursing Facility (SNF)), and others. This information was obtained from the electronic health record (EHR), where it was documented in a structured survey form that included an embedded open-ended field completed as part of routine clinical documentation. These data were extracted via retrospective chart review of the EHR.

#### Advanced Care Plan (ACP)

As a dichotomous variable (yes/no), it indicates whether the ACP has been discussed or not. The ACP is a process that involves making decisions about the medical care and treatments a patient would like to receive in the future, particularly in the event that they become unable to communicate or make decisions for themselves.

#### The Medical Orders for Life-Sustaining Treatment (MOLST) form

A binary variable that indicates whether the Medical Orders for Life-Sustaining Treatment (MOLST) form has been completed or not. The MOLST form is a medical document that provides specific medical orders regarding life-sustaining treatments for patients with advanced illnesses. In some U.S. states, a similar form called the Physician Orders for Life-Sustaining Treatment (POLST) is used. Both MOLST and POLST are provider-signed medical orders that translate a patient’s care preferences into actionable instructions for healthcare professionals. Unlike advanced directives, POLST/MOLST forms are immediately applicable across care settings and are designed for individuals with serious illness or advanced frailty who are at risk of a medical emergency. These forms typically include specific directives regarding interventions such as resuscitation, intubation, and hospitalization.

#### Co-morbidity risk

As a dichotomous variable (yes/no), it indicates whether the patient or anyone in their household has an at-risk co-morbidity, defined as a medical condition associated with increased vulnerability to adverse health outcomes, such as immunocompromised status. immunocompromised).

#### Palliative Performance Scale (PPS)

The PPS scale is used to assess functional status in palliative care settings. It helps healthcare providers evaluate the progression of disease, prognosis, and appropriate level of care for individuals with serious illnesses, particularly those with life-limiting conditions (ranging from 0 to 100) [21]. The PPS is a reliable and valid tool, with a mean Cohen’s kappa of 0.67–0.71. Specifically in terms of validity, although its application may vary across clinicians, it has been widely recognized as a valuable clinical assessment tool in palliative care. It has also been incorporated into routine clinical practice in many palliative care settings [21]. PPS scores were available only for patients who received HPC, as the PPS assessment was conducted as part of HPC intake and follow-up clinical evaluation.

#### Engaging in home-based palliative care (HPC)

It was defined as having received at least one home visit from the HPC team. This includes any in-person assessment or consultation conducted in the patient’s home as part of the HPC program.

### Data analysis

All the data were analyzed by SAS (ver.9.4, SAS Institute Inc, 2019). Continuous data were presented in terms of mean ± standard deviation for normally distributed variables or median and compared using Student’s T-test or Mann–Whitney U test based on whether or not normal distribution was assumed. Chi-square tests were used for comparisons of categorical variables between groups. Time to first HPC visit was analyzed using a Cox proportional hazards regression model. All factors selected above were included in the model. The Cox model requires the proportional hazard assumption to be verified. Cox’s regression was first used to examine all of the demographics/others associated with the first visit of the HPC service. All selected covariates were included in the final adjusted model. All tests were two-tailed, and the type I error level was set at 0.05.

## Results

Among 567 cases, 419 (74%) received HPC, while 148 (26%) did not receive HPC despite a referral. The average age of individuals who received HPC (*n* = 419) did not differ significantly from the mean age of 84.8 years in those who did not receive HPC despite being referred (*n* = 148; mean age = 84.4; *p* = 0.65). Approximately two-thirds of the participants were female, with 60% (*n* = 253) of those who received HPC and 64% (*n* = 94) of those who did not receive HPC being women. There was no statistically significant difference in gender distribution between the two groups, regardless of whether they received HPC (*p* = 0.50). About half of them utilized Medicare Part B for their insurance. There was no statistically significant difference observed between individuals with Medicare Part B (*n* = 239, 57%) who received HPC, and those (*n* = 80, 54%) who did not (*p* = 0.53). However, within the group of individuals who received HPC, there was a notable tendency for referrals from hospitals (*n* = 311, 74%) compared to those from primary care providers (*n* = 88, 21%) or other sources (*n* = 20, 4.8%). This discrepancy resulted in statistically significant differences in the referral patterns. (*p* < 0,01, Table 1).

**Table 1:** Participant demographic and referral characteristics

Variables		Those who received HPC (*n* = 419)	Those who did not receive HPC despite a referral (*n* = 148)	Significance	
Mean Age		84.8 (SD = 8.7)	84.4 (SD = 8.6)		0.65
Gender	Male	166 (39.6%)	54 (36.5%)		0.50
	Female	253 (60.4%)	94 (63.5%)		
Referral type	Hospital	311 (74.2%)	87 (58.8%)		< 0.01*
	Primary Care	88 (21%)	50 (33.8%)		
	Others	20 (4.8%)	11 (7.4%)		
Insurance	Medicare Part B	80 (54.1%)	239 (57%)		0.53
	Others	68 (46%)	180 (43%)		

*HPC* Home-based Palliative Care, *SD* standard deviation

* The race variable has approximately 83% missing data, so it was not included in this table

### Reasons for non-receipt of HPC following referral

Among those who were referred but did not receive HPC services, 25% reported being unable to access clinical care, 24% stated that their referral was either canceled or not provided, and 8% cited a lack of payer sources (insurance). Additionally, 12% were transferred to other facilities before their first visit (such as hospitals), 7% to other home health agencies, and 25% to skilled nursing facilities. Finally, 16% refused the services.

### Characteristics and impact on PPS for those who received HPC

Among those who received HPC (*n* = 419), two-thirds of participants (61%, *n* = 257) completed the MOLST form, and none changed their insurance during the study period. Additionally, 61% (*n* = 257) had an Advance Care Plan, and 49% (*n* = 207) had a Do Not Resuscitate (DNR) order in place before the consultation. Furthermore, 49% received spiritual or social work support, and 12% (*n* = 53) were referred to hospice care only. About 95% of respondents answered the question about whether the patient or anyone in the household has an at-risk comorbidity, as a health condition that could increase the risk of worse health outcomes, such as being immunocompromised.

During the study period (2022–2023), the majority of participants only utilized HPC once, with 260 individuals accounting for 62%. The frequency of HPC usage ranged from 1 to 10 times during this study period. In the comparison between individuals (*n* = 260) who utilized HPC once and those (*n* = 159) who used it two or more times, it was observed that those with more than two instances of usage exhibited higher PPS scores (mean = 47.7) than those who used HPC only once (mean = 46.2). However, this difference was not statistically significant (z = 0.7, *p* = 0.48). These findings reflect differences in PPS values between patients with varying levels of HPC utilization rather than changes in PPS over time. Regarding the different referral types (hospitals/medical centers, primary care providers/physicians’ offices, and others), the average PPS score was lower in the “other” group (44.3), compared to hospital referrals (47.6) and physician office referrals (47.7). However, these differences were not statistically significant (*p* = 0.27).

The most common primary diagnoses among participants who used HPC were cancer (16%, *n* = 67), neurological disorders (15%, *n* = 62), COPD and other respiratory diseases (11%, *n* = 48), mental health disorders (15%, *n* = 62), urinary and reproductive system diseases (7%, *n* = 30), and other conditions (30%, *n* = 127). Specifically, 5% of participants had Parkinson’s disease (*n* = 23), while 9% had dementia, Alzheimer’s, or cognitive decline (*n* = 37). Regarding the PPS scores, there was no significant difference in PPS scores between patients with cancer (mean = 49.6) and those without cancer (mean = 48.6, z = 0.35, *p* = 0.71). However, patients with dementia, Alzheimer’s disease, or cognitive decline had significantly lower PPS scores (mean = 43.1) compared to those without these conditions (mean = 49.3, z = -2.4, *p* = 0.02).

Individuals who completed the MOLST form had lower PPS scores (mean = 50.3 for the group that completed the MOLST form vs. mean = 47.7 for those who did not), but the difference was not statistically significant (z = 1.71, *p* = 0.09). In contrast, individuals with a DNR order in place had significantly lower PPS scores (mean = 51.5) compared to those without a DNR order (mean = 45.8) (z = -4.3, *p* < 0.01).

### Time to receive the first HPC service

**Fig. 1:**
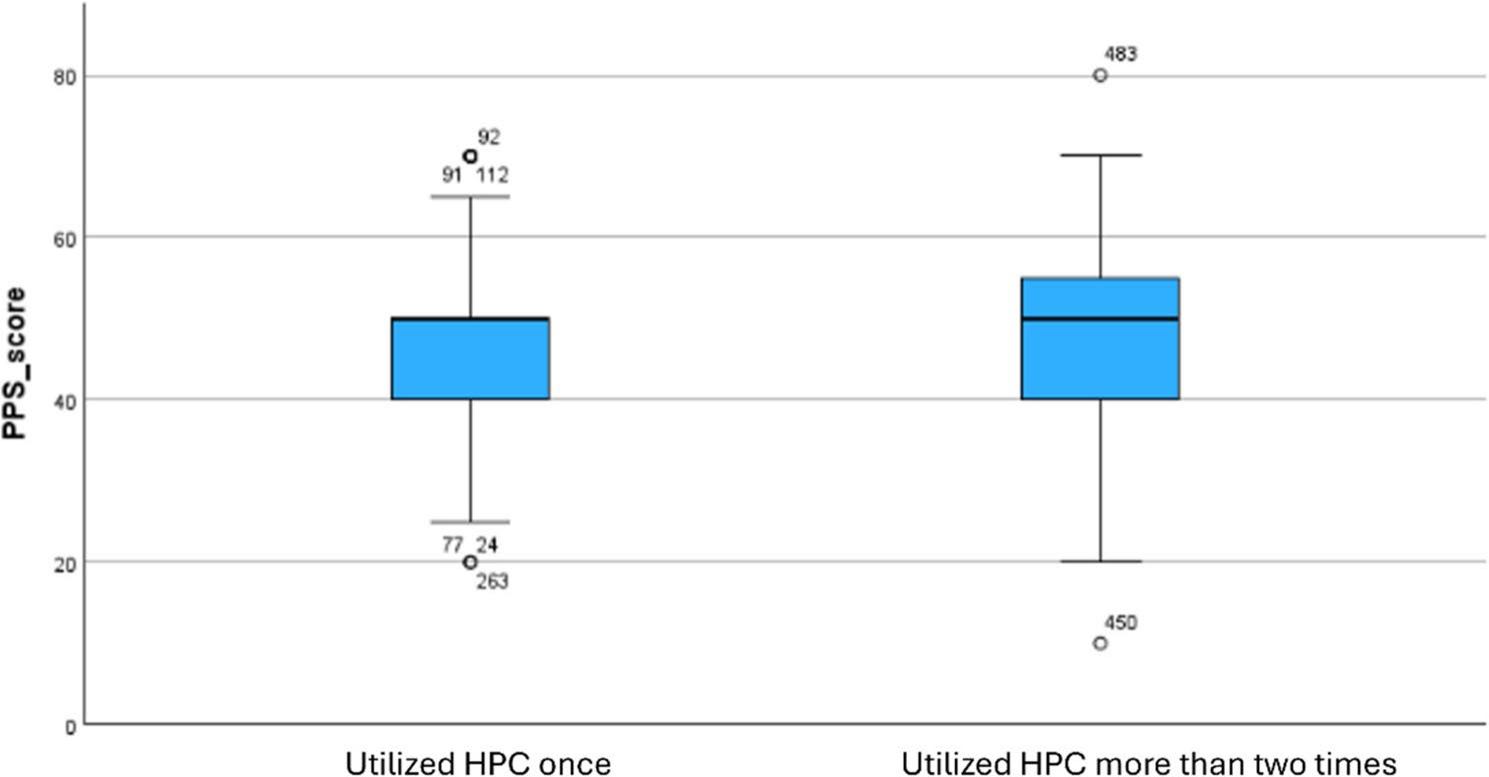
PPS scores between individuals who utilized HPC once and those who used it more than two times. Note: Palliative Performance Scale (PPS); Home-based Palliative Care (HPC)

**Fig. 2:**
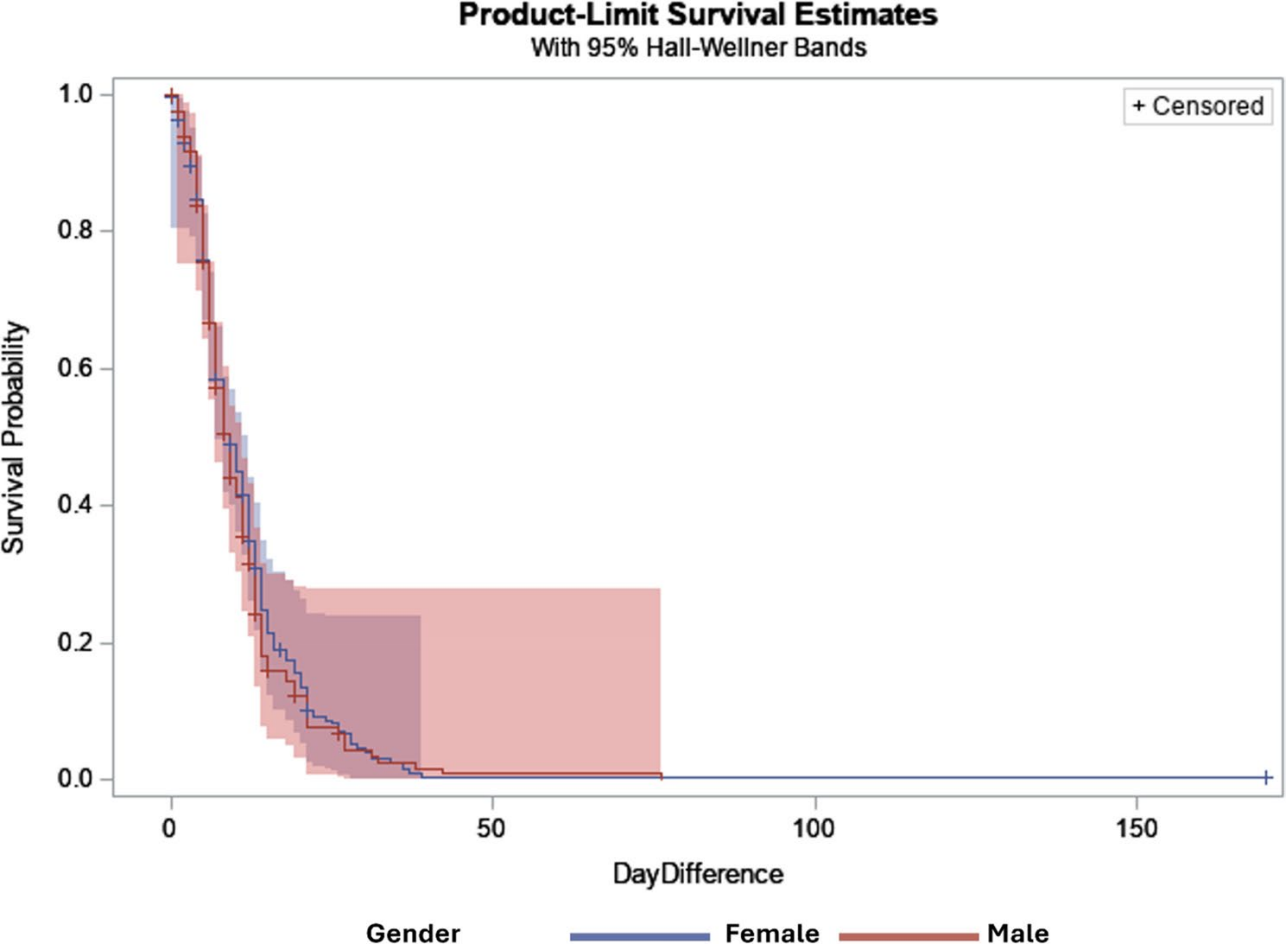
Time to receive the first HPC, categorized by gender. Note: Home-based Palliative Care (HPC)

**Fig. 3:**
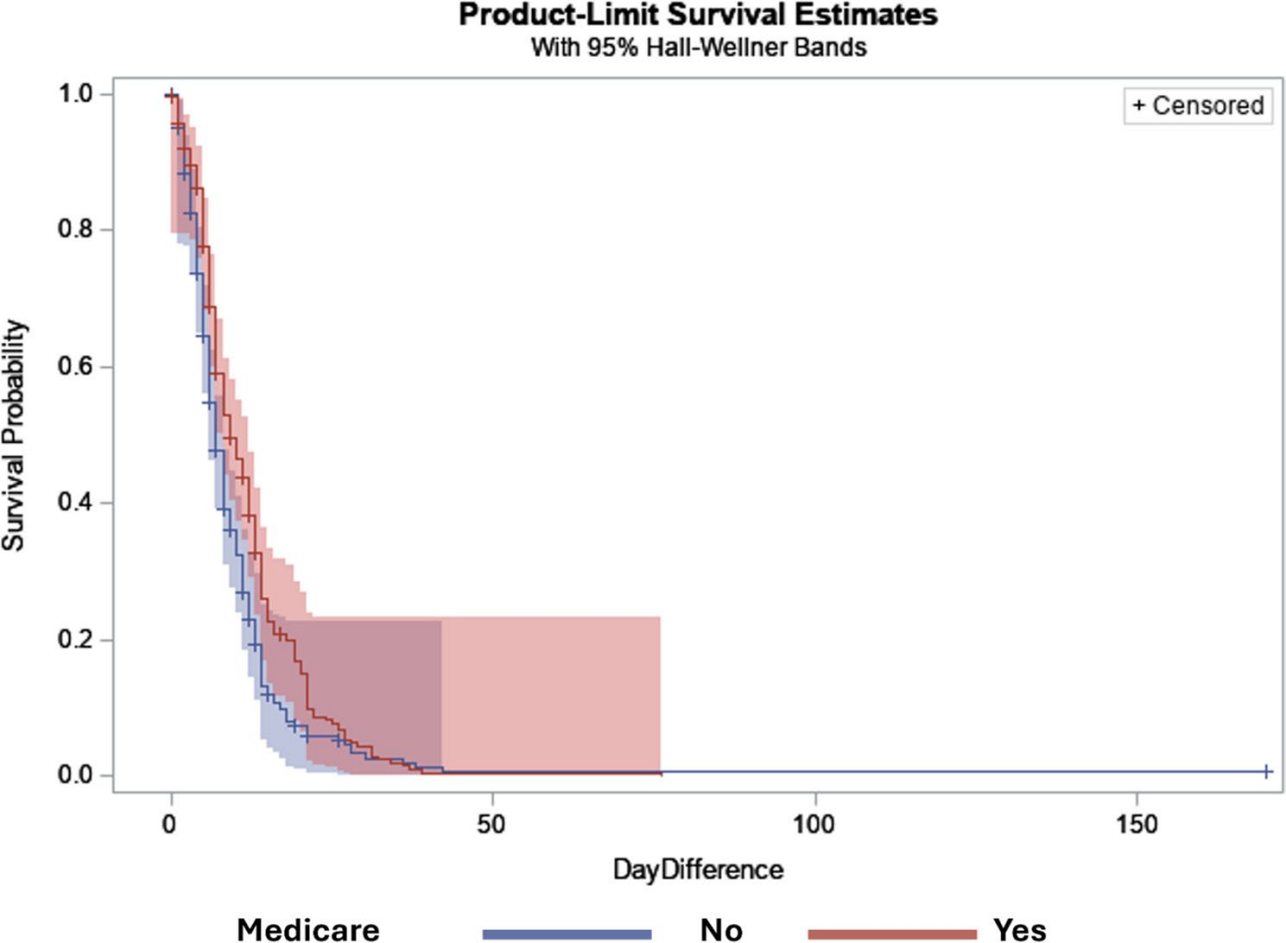
Time to receive the first HPC, categorized by Medicare. Note: Home-based Palliative Care (HPC)

**Fig. 4:**
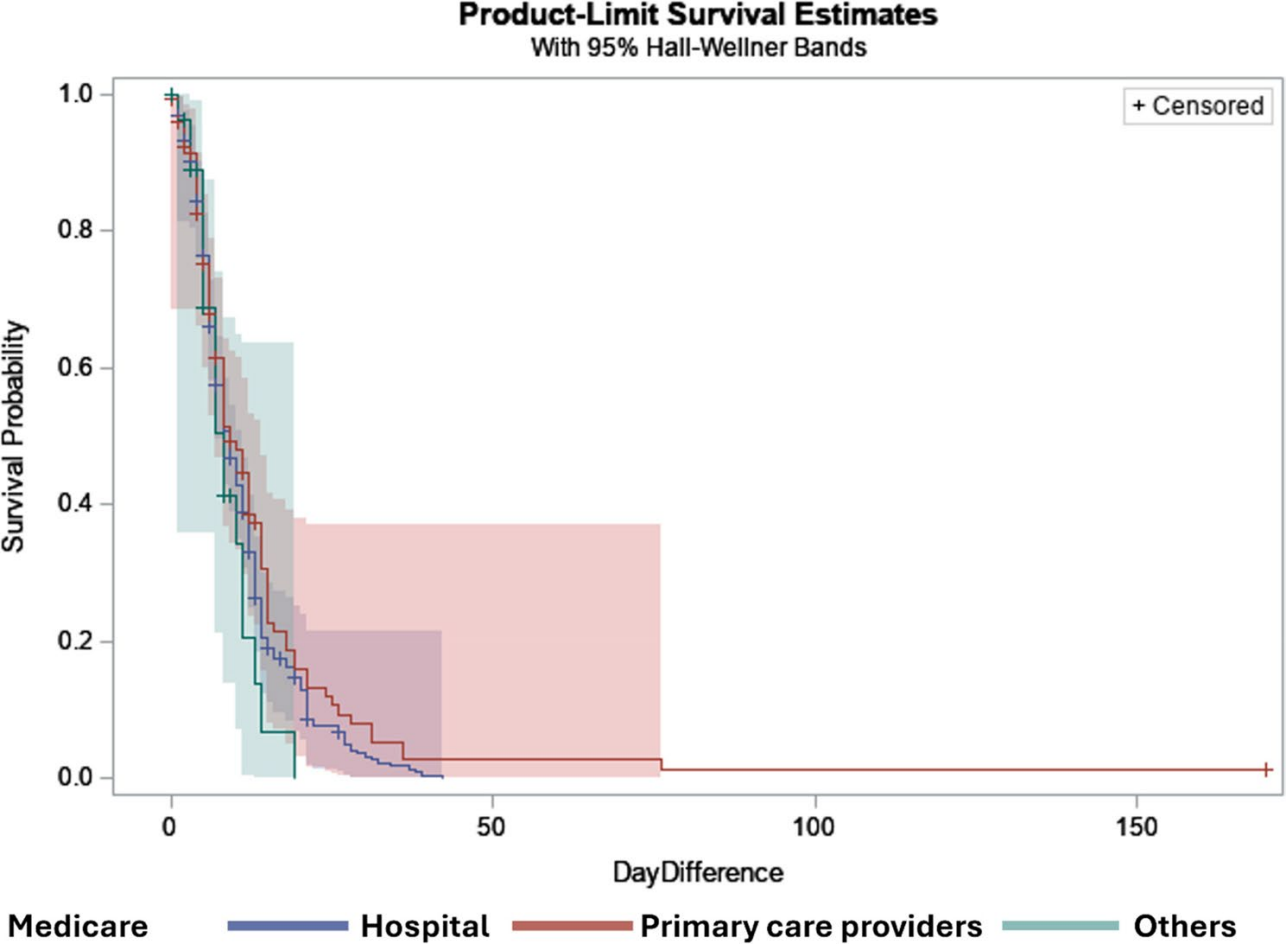
Time to receive the first HPC, categorized by referral types. Note: Home-based Palliative Care (HPC)

On average, patients who received HPC started the service 7 days after their referral. For those who did not receive HPC, the decision was made about 9 days after the referral. Among those who received HPC, there was no difference between females and males in terms of the first time receiving HPC (*p* = 0.50, Fig. 1). However, those covered by Medicare Part B experienced a delay in receiving the initial HPC care, compared to those without Medicare Part B (Fig. 2). It indicates a statistically significant difference in the timing of the first HPC care based on individuals having Medicare Part B as their payor or not (*p* < 0.01, Fig. 3). Regarding three referral types ((1) hospital, (2) primary care providers, and (3) others), there was a statistically significant distinction in the timing of the initial HPC care after the referral (*p* = 0.03, Fig. 4).

## Discussion

This study examined the factors influencing the utilization of home palliative care (HPC) among older adults in a cohort of 567 individuals, with 419 receiving HPC and 148 not receiving it despite a referral. Overall, demographic characteristics such as age (*p* = 0.65) and gender (*p* = 0.50) were not significantly associated with HPC utilization, and insurance type, specifically Medicare Part B, did not differ significantly between groups in terms of uptake. However, patients covered under Medicare Part B experienced significantly longer delays in receiving their first HPC consultation (*p* < 0.01), suggesting that insurance structure may influence timeliness rather than initial access. These delays may stem from administrative challenges, authorization issues, or specific limitations within Medicare Part B coverage [22, 23].

To better interpret these findings, results can be contextualized using Levesque’s framework encompassing approachability, acceptability, availability, accommodation, affordability, and appropriateness [20]. Within this framework, delays associated with Medicare Part B most strongly reflect barriers in affordability and accommodation, where reimbursement policies and administrative processes may hinder timely service delivery. The Medicare system structure likely contributes to these barriers, as HPC is typically reimbursed under the Physician Fee Schedule, which emphasizes individual clinician visits and may inadequately support interdisciplinary care, care coordination, and home-based service delivery [24–26]. This fragmented reimbursement model may increase administrative burden and contribute to delays in care initiation.

Approachability and system awareness are reflected in referral patterns, which demonstrated that most HPC referrals originated from hospitals, followed by primary care providers and other sources. Patients referred from hospital settings accessed HPC more quickly, suggesting stronger referral infrastructure, greater familiarity with HPC services, and more streamlined processes in these settings. In contrast, lower referral rates and slower access from primary care may indicate gaps in awareness or integration of HPC services. Although physicians typically initiate referrals, the referral process is interdisciplinary, often involving social workers, case managers, and discharge planners, emphasizing the importance of coordinated systems in facilitating access [27]. Targeted education and workflow integration across all members of the care team—not only physicians—may improve approachability and streamline referral pathways [28–32].

Availability remains a key structural barrier, particularly in suburban and rural settings where this study was conducted. Limited workforce capacity, geographic dispersion, and logistical challenges in delivering home-based services may constrain access to HPC, even when referrals are made [17, 33, 34]. Among patients who did not receive HPC, lack of service availability and canceled or unfulfilled referrals were among the most commonly reported barriers, underscoring the importance of service capacity over purely financial limitations.

Accommodation and appropriateness are further highlighted in patterns of utilization among specific patient populations. Patients with cognitive impairment were less likely to receive HPC and had lower PPS scores, suggesting delayed referral and more advanced functional decline at the time of access [4–7, 10, 15, 19, 35–38]. This pattern may reflect ongoing uncertainty regarding the appropriateness of HPC for non-cancer diagnoses, as well as logistical challenges in delivering home-based services to cognitively impaired individuals. Furthermore, many patients with dementia reside in institutional settings, where HPC may be less applicable or less frequently utilized, thereby further shaping observed utilization patterns [30–32]. Diagnostic trends showed that cancer, neurological disorders, COPD, mental health conditions, Parkinson’s disease, and dementia/Alzheimer’s disease were among the most common conditions in HPC users; however, despite this clinical diversity, disparities in access persist—particularly for non-cancer diagnoses—reinforcing concerns regarding appropriateness and equity in referral practices.

Patterns of service utilization further reflect complexity in access and care delivery. Most patients utilized HPC only once during the study period, which may indicate early-stage referral, limited follow-up capacity, or variability in patient needs and care trajectories. Caregiver availability likely plays a critical role, as informal caregivers are often essential to sustaining home-based care, influencing both initiation and continuation of HPC services [39, 40]. Patients with more frequent encounters had higher PPS scores; however, this represents an association between functional status and utilization rather than longitudinal improvement, as PPS was measured at each encounter and not tracked as a continuous trajectory.

Acceptability and broader sociocultural factors may also influence HPC utilization. Cultural perceptions of palliative care, stigma, and misconceptions distinguishing palliative care from hospice remain persistent barriers [16, 27]. Limited integration of HPC referral processes within electronic health records and variability in provider knowledge may further reduce effective access, particularly in non-hospital settings [39, 41–44].

Finally, although many patients express a preference for receiving care at home due to comfort, familiarity, and independence, system-level barriers across multiple dimensions of access limit the realization of these preferences. Prior literature demonstrates that HPC is associated with improved symptom management, reduced healthcare utilization, and higher patient and caregiver satisfaction, highlighting the importance of addressing access barriers to align care delivery with patient goals and optimize outcomes [10, 15, 35–38].

### Limitations

A key limitation of this study is its reliance on data from a single healthcare facility, with a relatively small sample size collected over a one-year period. Additionally, the facility primarily serves suburban and rural areas, which may limit the generalizability of the results to other settings or populations. As the facility is not part of a larger integrated healthcare system, the dataset includes only patients who received HPC within the organization. Consequently, the study may not capture individuals who accessed palliative care services through other providers. Furthermore, race and ethnicity data were missing for more than 80% of participants and therefore could not be included in the analysis, limiting the ability to examine potential disparities in HPC utilization. Furthermore, the dataset lacks detailed information regarding the referral process, including whether referrals were consistently documented within the Electronic Health Records (EHR) system. In addition, information on the presence of a caregiver or informal caregiving support was not available in the dataset, limiting the ability to account for this important contextual factor in home-based palliative care. Also, external factors such as health insurance coverage may have influenced access to services and patient engagement beyond what is reflected in our findings. Another limitation of this study is the potential variability and inconsistency in PPS scores, as they were documented by different providers during routine clinical care. This may have affected inter-rater reliability and could have influenced the study findings related to PPS. Future studies should include larger samples from multiple sites to address current HPC challenges. Additionally, qualitative research can offer deeper insights into the specific challenges of accessing HPC from the perspectives of families/patients, providers, and health policymakers. In future studies, it would be valuable to explore concealed structural disparities in accessing home palliative care within our healthcare system and their ramifications.

## Conclusions

This study highlights that while gender, race, and age did not significantly differ between those who received home palliative care (HPC) services and those who did not, disparities emerged based on insurance, and referral sources. Barriers such as limited access to clinical care canceled referrals, and patient refusal were key factors preventing service receipt. The findings offer valuable insight into the practical benefits of HPC and how it may improve access to care for individuals who are referred to but ultimately do not receive services. Recognizing and addressing disparities in access to HPC, along with understanding the deeper impact of these systemic inequities, is critical to promoting more equitable delivery of palliative care services.

## Data Availability

The data used in this study are not publicly available due to restrictions imposed by the Visiting Nurse Association (VNA). Access to the data may be considered upon reasonable request and with approval from the VNA.
